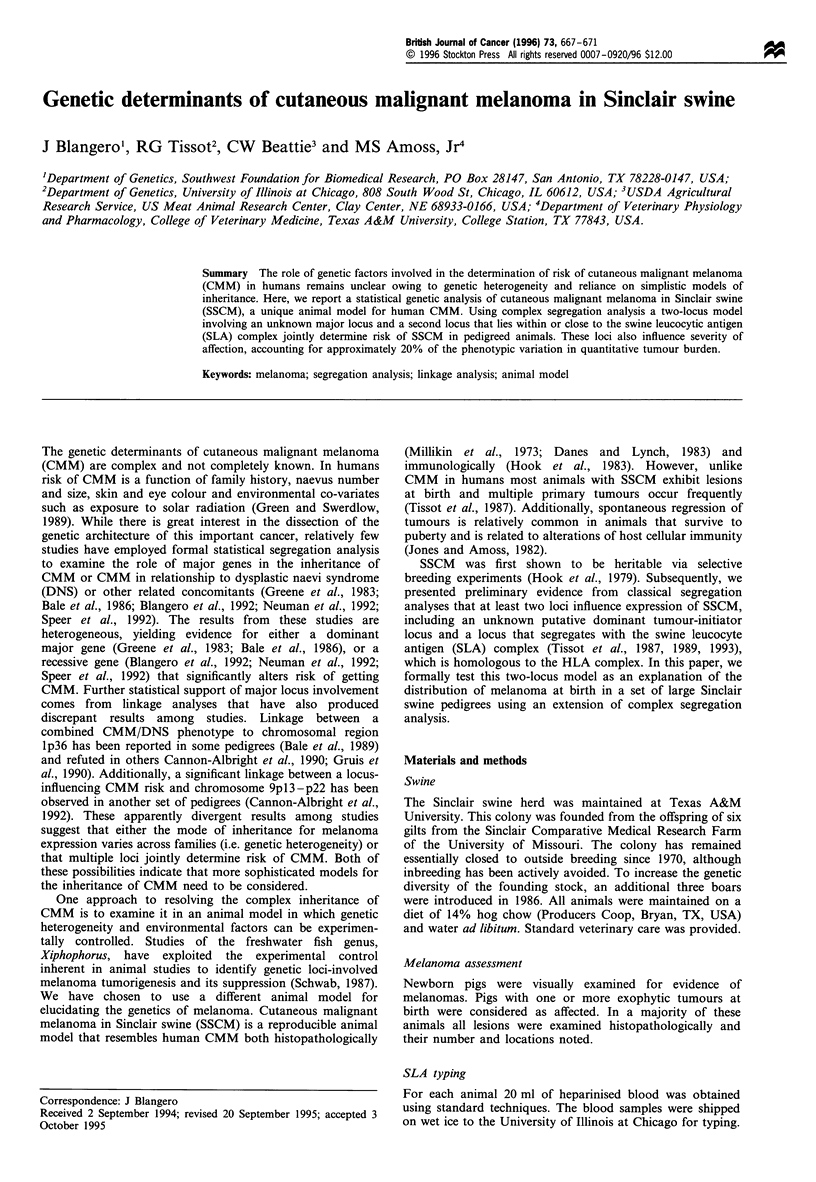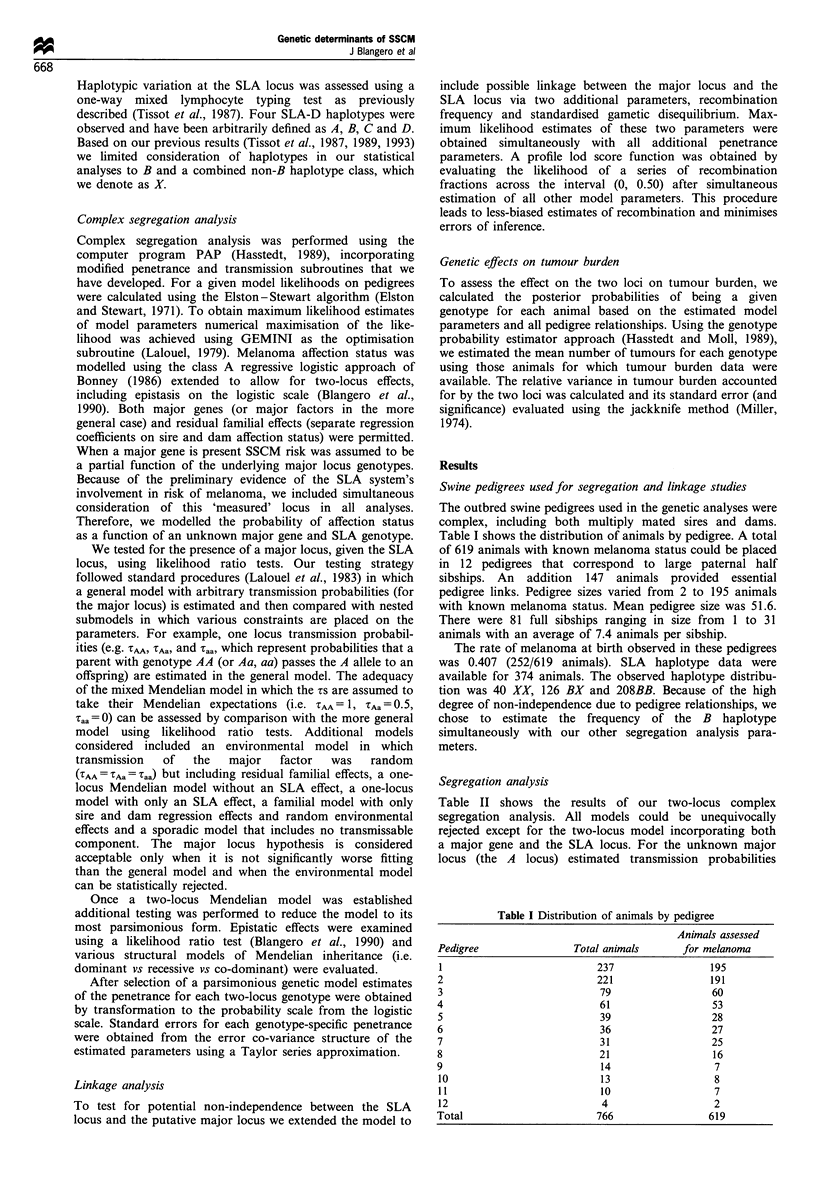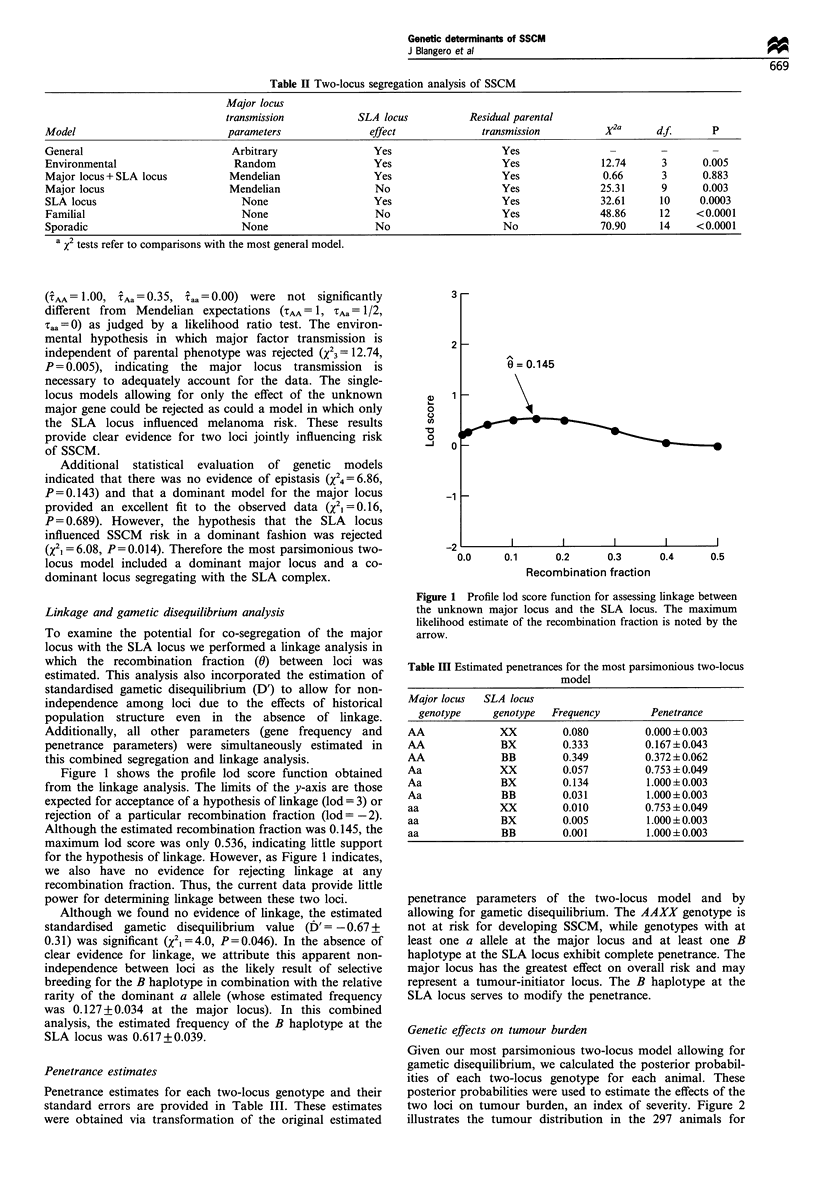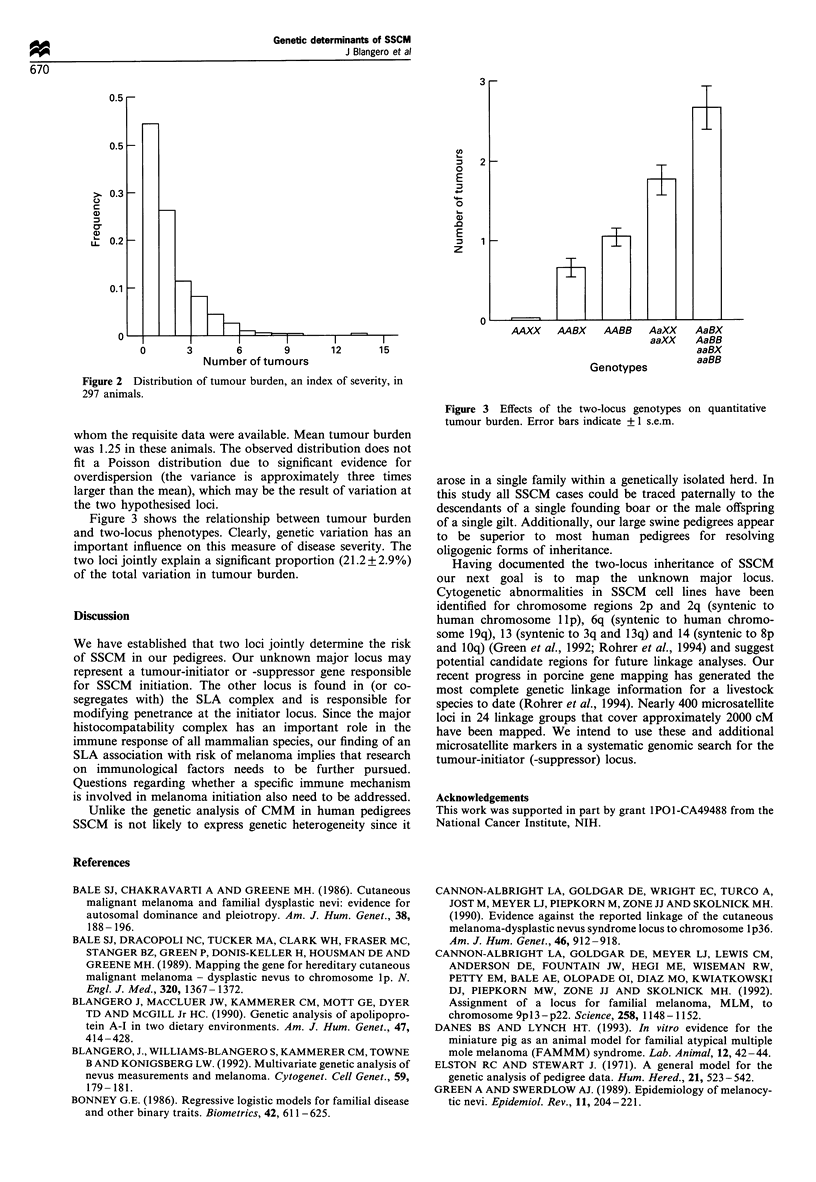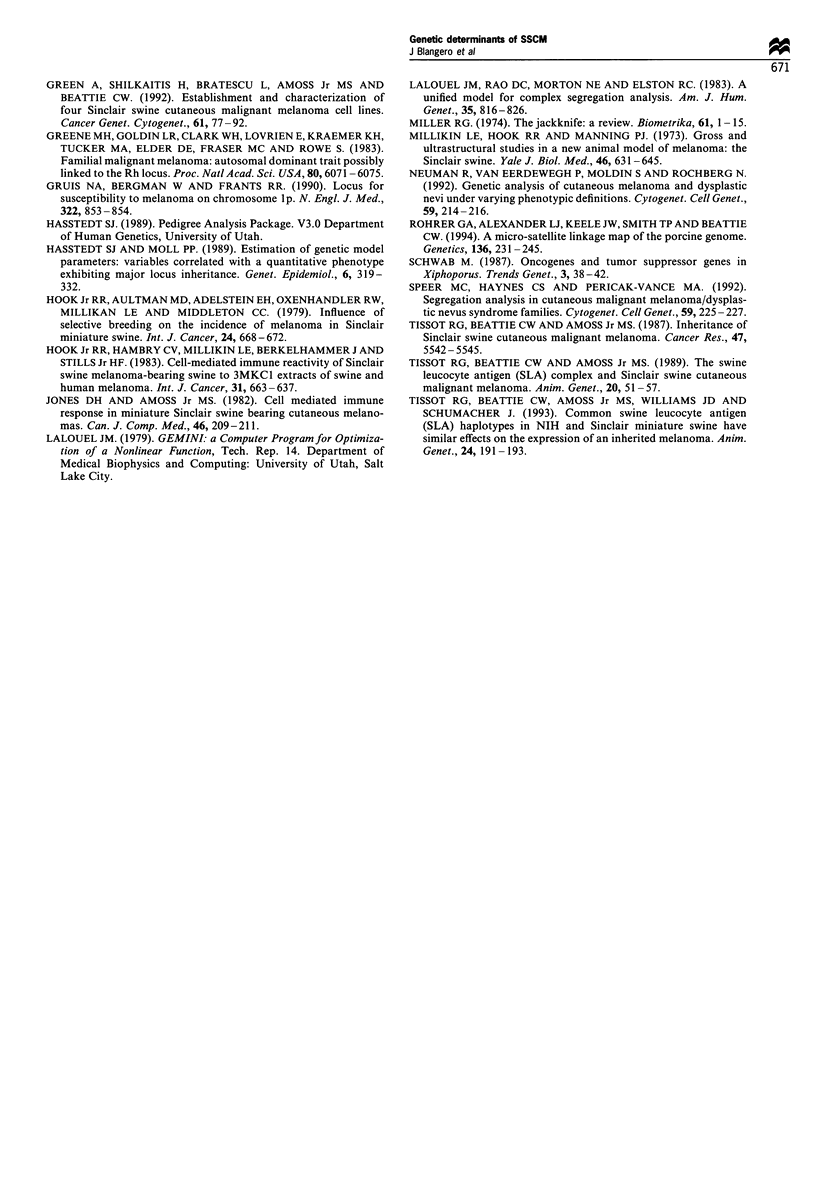# Genetic determinants of cutaneous malignant melanoma in Sinclair swine.

**DOI:** 10.1038/bjc.1996.116

**Published:** 1996-03

**Authors:** J. Blangero, R. G. Tissot, C. W. Beattie, M. S. Amoss

**Affiliations:** Department of Genetics, Southwest Foundation for Biomedical Reserach, San Antonio, TX 78228-0147, USA.

## Abstract

The role of genetic factors involved in the determination of risk of cutaneous malignant melanoma (CMM) in humans remains unclear owing to genetic heterogeneity and reliance on simplistic models of inheritance. Here, we report a statistical genetic analysis of cutaneous malignant melanoma in Sinclair swine (SSCM), a unique animal model for human CMM. Using complex segregation analysis a two-locus model involving an unknown major locus and a second locus that lies within or close to the swine leukocyte antigen (SLA) complex jointly determine risk of SSCM in pedigreed animals. These loci also influence severity of affection, accounting for approximately 20% of the phenotypic variation in quantitative tumour burden.


British Journal of Cancer (1996) 73, 667–671
© 1996 Stockton Press All rights reserved 0007–0920/96 $12.00

### Genetic determinants of cutaneous malignant melanoma in Sinclair swine

J Blangero[1], RG Tissot[2], CW Beattie[3] and MS Amoss, Jr[4]

[1]*Department of Genetics, Southwest Foundation for Biomedical Research, PO Box 28147, San Antonio, TX 78228-0147, USA;* [2]*Department of Genetics, University of Illinois at Chicago, 808 South Wood St, Chicago, IL 60612, USA;* [3]*USDA Agricultural Research Service, US Meat Animal Research Center, Clay Center, NE 68933-0166, USA;* [4]*Department of Veterinary Physiology and Pharmacology, College of Veterinary Medicine, Texas A&M University, College Station, TX 77843, USA.*

**Summary** The role of genetic factors involved in the determination of risk of cutaneous malignant melanoma (CMM) in humans remains unclear owing to genetic heterogeneity and reliance on simplistic models of inheritance. Here, we report a statistical genetic analysis of cutaneous malignant melanoma in Sinclair swine (SSCM), a unique animal model for human CMM. Using complex segregation analysis a two-locus model involving an unknown major locus and a second locus that lies within or close to the swine leucocytic antigen (SLA) complex jointly determine risk of SSCM in pedigreed animals. These loci also influence severity of affection, accounting for approximately 20% of the phenotypic variation in quantitative tumour burden.

**Keywords:** melanoma; segregation analysis; linkage analysis; animal model

The genetic determinants of cutaneous malignant melanoma (CMM) are complex and not completely known. In humans risk of CMM is a function of family history, naevus number and size, skin and eye colour and environmental co-variates such as exposure to solar radiation (Green and Swerdlow, 1989). While there is great interest in the dissection of the genetic architecture of this important cancer, relatively few studies have employed formal statistical segregation analysis to examine the role of major genes in the inheritance of CMM or CMM in relationship to dysplastic naevi syndrome (DNS) or other related concomitants (Greene *et al.*, 1983; Bale *et al.*, 1986; Blangero *et al.*, 1992; Neuman *et al.*, 1992; Speer *et al.*, 1992). The results from these studies are heterogeneous, yielding evidence for either a dominant major gene (Greene *et al.*, 1983; Bale *et al.*, 1986), or a recessive gene (Blangero *et al.*, 1992; Neuman *et al.*, 1992; Speer *et al.*, 1992) that significantly alters risk of getting CMM. Further statistical support of major locus involvement comes from linkage analyses that have also produced discrepant results among studies. Linkage between a combined CMM/DNS phenotype to chromosomal region 1p36 has been reported in some pedigrees (Bale *et al.*, 1989) and refuted in others Cannon-Albright *et al.*, 1990; Gruis *et al.*, 1990). Additionally, a significant linkage between a locus-influencing CMM risk and chromosome 9p13–p22 has been observed in another set of pedigrees (Cannon-Albright *et al.*, 1992). These apparently divergent results among studies suggest that either the mode of inheritance for melanoma expression varies across families (i.e. genetic heterogeneity) or that multiple loci jointly determine risk of CMM. Both of these possibilities indicate that more sophisticated models for the inheritance of CMM need to be considered.

One approach to resolving the complex inheritance of CMM is to examine it in an animal model in which genetic heterogeneity and environmental factors can be experimentally controlled. Studies of the freshwater fish genus, *Xiphophorus*, have exploited the experimental control inherent in animal studies to identify genetic loci-involved melanoma tumorigenesis and its suppression (Schwab, 1987). We have chosen to use a different animal model for elucidating the genetics of melanoma. Cutaneous malignant melanoma in Sinclair swine (SSCM) is a reproducible animal model that resembles human CMM both histopathologically (Millikin *et al.*, 1973; Danes and Lynch, 1983) and immunologically (Hook *et al.*, 1983). However, unlike CMM in humans most animals with SSCM exhibit lesions at birth and multiple primary tumours occur frequently (Tissot *et al.*, 1987). Additionally, spontaneous regression of tumours is relatively common in animals that survive to puberty and is related to alterations of host cellular immunity (Jones and Amoss, 1982).

SSCM was first shown to be heritable via selective breeding experiments (Hook *et al.*, 1979). Subsequently, we presented preliminary evidence from classical segregation analyses that at least two loci influence expression of SSCM, including an unknown putative dominant tumour-initiator locus and a locus that segregates with the swine leucocyte antigen (SLA) complex (Tissot *et al.*, 1987, 1989, 1993), which is homologous to the HLA complex. In this paper, we formally test this two-locus model as an explanation of the distribution of melanoma at birth in a set of large Sinclair swine pedigrees using an extension of complex segregation analysis.

### Materials and methods

### *Swine*

The Sinclair swine herd was maintained at Texas A&M University. This colony was founded from the offspring of six gilts from the Sinclair Comparative Medical Research Farm of the University of Missouri. The colony has remained essentially closed to outside breeding since 1970, although inbreeding has been actively avoided. To increase the genetic diversity of the founding stock, an additional three boars were introduced in 1986. All animals were maintained on a diet of 14% hog chow (Producers Coop, Bryan, TX, USA) and water *ad libitum*. Standard veterinary care was provided.

### *Melanoma assessment*

Newborn pigs were visually examined for evidence of melanomas. Pigs with one or more exophytic tumours at birth were considered as affected. In a majority of these animals all lesions were examined histopathologically and their number and locations noted.

### *SLA typing*

For each animal 20 ml of heparinised blood was obtained using standard techniques. The blood samples were shipped on wet ice to the University of Illinois at Chicago for typing.

Correspondence: J Blangero
Received 2 September 1994; revised 20 September 1995; accepted 3 October 1995

Haplotypic variation at the SLA locus was assessed using a one-way mixed lymphocyte typing test as previously described (Tissot *et al.*, 1987). Four SLA-D haplotypes were observed and have been arbitrarily defined as *A*, *B*, *C* and *D*. Based on our previous results (Tissot *et al.*, 1987, 1989, 1993) we limited consideration of haplotypes in our statistical analyses to *B* and a combined non-*B* haplotype class, which we denote as *X*.

### *Complex segregation analysis*

Complex segregation analysis was performed using the computer program PAP (Hasstedt, 1989), incorporating modified penetrance and transmission subroutines that we have developed. For a given model likelihoods on pedigrees were calculated using the Elston–Stewart algorithm (Elston and Stewart, 1971). To obtain maximum likelihood estimates of model parameters numerical maximisation of the likelihood was achieved using GEMINI as the optimisation subroutine (Lalouel, 1979). Melanoma affection status was modelled using the class A regressive logistic approach of Bonney (1986) extended to allow for two-locus effects, including epistasis on the logistic scale (Blangero *et al.*, 1990). Both major genes (or major factors in the more general case) and residual familial effects (separate regression coefficients on sire and dam affection status) were permitted. When a major gene is present SSCM risk was assumed to be a partial function of the underlying major locus genotypes. Because of the preliminary evidence of the SLA system's involvement in risk of melanoma, we included simultaneous consideration of this 'measured' locus in all analyses. Therefore, we modelled the probability of affection status as a function of an unknown major gene and SLA genotype.

We tested for the presence of a major locus, given the SLA locus, using likelihood ratio tests. Our testing strategy followed standard procedures (Lalouel *et al.*, 1983) in which a general model with arbitrary transmission probabilities (for the major locus) is estimated and then compared with nested submodels in which various constraints are placed on the parameters. For example, one locus transmission probabilities (e.g. $\tau_{AA}$, $\tau_{Aa}$, and $\tau_{aa}$, which represent probabilities that a parent with genotype *AA* (or *Aa*, *aa*) passes the *A* allele to an offspring) are estimated in the general model. The adequacy of the mixed Mendelian model in which the $\tau$s are assumed to take their Mendelian expectations (i.e. $\tau_{AA}=1$, $\tau_{Aa}=0.5$, $\tau_{aa}=0$) can be assessed by comparison with the more general model using likelihood ratio tests. Additional models considered included an environmental model in which transmission of the major factor was random ($\tau_{AA}=\tau_{Aa}=\tau_{aa}$) but including residual familial effects, a one-locus Mendelian model without an SLA effect, a one-locus model with only an SLA effect, a familial model with only sire and dam regression effects and random environmental effects and a sporadic model that includes no transmissable component. The major locus hypothesis is considered acceptable only when it is not significantly worse fitting than the general model and when the environmental model can be statistically rejected.

Once a two-locus Mendelian model was established additional testing was performed to reduce the model to its most parsimonious form. Epistatic effects were examined using a likelihood ratio test (Blangero *et al.*, 1990) and various structural models of Mendelian inheritance (i.e. dominant *vs* recessive *vs* co-dominant) were evaluated.

After selection of a parsimonious genetic model estimates of the penetrance for each two-locus genotype were obtained by transformation to the probability scale from the logistic scale. Standard errors for each genotype-specific penetrance were obtained from the error co-variance structure of the estimated parameters using a Taylor series approximation.

### *Linkage analysis*

To test for potential non-independence between the SLA locus and the putative major locus we extended the model to include possible linkage between the major locus and the SLA locus via two additional parameters, recombination frequency and standardised gametic disequilibrium. Maximum likelihood estimates of these two parameters were obtained simultaneously with all additional penetrance parameters. A profile lod score function was obtained by evaluating the likelihood of a series of recombination fractions across the interval (0, 0.50) after simultaneous estimation of all other model parameters. This procedure leads to less-biased estimates of recombination and minimises errors of inference.

### *Genetic effects on tumour burden*

To assess the effect on the two loci on tumour burden, we calculated the posterior probabilities of being a given genotype for each animal based on the estimated model parameters and all pedigree relationships. Using the genotype probability estimator approach (Hasstedt and Moll, 1989), we estimated the mean number of tumours for each genotype using those animals for which tumour burden data were available. The relative variance in tumour burden accounted for by the two loci was calculated and its standard error (and significance) evaluated using the jackknife method (Miller, 1974).

### Results

### *Swine pedigrees used for segregation and linkage studies*

The outbred swine pedigrees used in the genetic analyses were complex, including both multiply mated sires and dams. Table I shows the distribution of animals by pedigree. A total of 619 animals with known melanoma status could be placed in 12 pedigrees that correspond to large paternal half sibships. An addition 147 animals provided essential pedigree links. Pedigree sizes varied from 2 to 195 animals with known melanoma status. Mean pedigree size was 51.6. There were 81 full sibships ranging in size from 1 to 31 animals with an average of 7.4 animals per sibship.

The rate of melanoma at birth observed in these pedigrees was 0.407 (252/619 animals). SLA haplotype data were available for 374 animals. The observed haplotype distribution was 40 *XX*, 126 *BX* and 208*BB*. Because of the high degree of non-independence due to pedigree relationships, we chose to estimate the frequency of the *B* haplotype simultaneously with our other segregation analysis parameters.

### *Segregation analysis*

Table II shows the results of our two-locus complex segregation analysis. All models could be unequivocally rejected except for the two-locus model incorporating both a major gene and the SLA locus. For the unknown major locus (the *A* locus) estimated transmission probabilities

**Table I** Distribution of animals by pedigree

| *Pedigree* | *Total animals* | *Animals assessed for melanoma* |
|---|---|---|
| 1 | 237 | 195 |
| 2 | 221 | 191 |
| 3 | 79 | 60 |
| 4 | 61 | 53 |
| 5 | 39 | 28 |
| 6 | 36 | 27 |
| 7 | 31 | 25 |
| 8 | 21 | 16 |
| 9 | 14 | 7 |
| 10 | 13 | 8 |
| 11 | 10 | 7 |
| 12 | 4 | 2 |
| Total | 766 | 619 |

**Table II** Two-locus segregation analysis of SSCM

| *Model* | *Major locus transmission parameters* | *SLA locus effect* | *Residual parental transmission* | $X^{2a}$ | *d.f.* | P |
|---|---|---|---|---|---|---|
| General | Arbitrary | Yes | Yes | – | – | – |
| Environmental | Random | Yes | Yes | 12.74 | 3 | 0.005 |
| Major locus + SLA locus | Mendelian | Yes | Yes | 0.66 | 3 | 0.883 |
| Major locus | Mendelian | No | Yes | 25.31 | 9 | 0.003 |
| SLA locus | None | Yes | Yes | 32.61 | 10 | 0.0003 |
| Familial | None | No | Yes | 48.86 | 12 | <0.0001 |
| Sporadic | None | No | No | 70.90 | 14 | <0.0001 |

[a] $\chi^2$ tests refer to comparisons with the most general model.

($\hat{\tau}_{AA}=1.00$, $\hat{\tau}_{Aa}=0.35$, $\hat{\tau}_{aa}=0.00$) were not significantly different from Mendelian expectations ($\tau_{AA}=1$, $\tau_{Aa}=1/2$, $\tau_{aa}=0$) as judged by a likelihood ratio test. The environmental hypothesis in which major factor transmission is independent of parental phenotype was rejected ($\chi^2_3=12.74$, $P=0.005$), indicating the major locus transmission is necessary to adequately account for the data. The single-locus models allowing for only the effect of the unknown major gene could be rejected as could a model in which only the SLA locus influenced melanoma risk. These results provide clear evidence for two loci jointly influencing risk of SSCM.

Additional statistical evaluation of genetic models indicated that there was no evidence of epistasis ($\chi^2_4=6.86$, $P=0.143$) and that a dominant model for the major locus provided an excellent fit to the observed data ($\chi^2_1=0.16$, $P=0.689$). However, the hypothesis that the SLA locus influenced SSCM risk in a dominant fashion was rejected ($\chi^2_1=6.08$, $P=0.014$). Therefore the most parsimonious two-locus model included a dominant major locus and a co-dominant locus segregating with the SLA complex.

### *Linkage and gametic disequilibrium analysis*

To examine the potential for co-segregation of the major locus with the SLA locus we performed a linkage analysis in which the recombination fraction ($\theta$) between loci was estimated. This analysis also incorporated the estimation of standardised gametic disequilibrium (D′) to allow for non-independence among loci due to the effects of historical population structure even in the absence of linkage. Additionally, all other parameters (gene frequency and penetrance parameters) were simultaneously estimated in this combined segregation and linkage analysis.

Figure 1 shows the profile lod score function obtained from the linkage analysis. The limits of the *y*-axis are those expected for acceptance of a hypothesis of linkage (lod = 3) or rejection of a particular recombination fraction (lod = −2). Although the estimated recombination fraction was 0.145, the maximum lod score was only 0.536, indicating little support for the hypothesis of linkage. However, as Figure 1 indicates, we also have no evidence for rejecting linkage at any recombination fraction. Thus, the current data provide little power for determining linkage between these two loci.

Although we found no evidence of linkage, the estimated standardised gametic disequilibrium value ($\hat{D}'=-0.67\pm 0.31$) was significant ($\chi^2_1=4.0$, $P=0.046$). In the absence of clear evidence for linkage, we attribute this apparent non-independence between loci as the likely result of selective breeding for the *B* haplotype in combination with the relative rarity of the dominant *a* allele (whose estimated frequency was $0.127\pm 0.034$ at the major locus). In this combined analysis, the estimated frequency of the *B* haplotype at the SLA locus was $0.617\pm 0.039$.

### *Penetrance estimates*

Penetrance estimates for each two-locus genotype and their standard errors are provided in Table III. These estimates were obtained via transformation of the original estimated penetrance parameters of the two-locus model and by allowing for gametic disequilibrium. The *AAXX* genotype is not at risk for developing SSCM, while genotypes with at least one *a* allele at the major locus and at least one *B* haplotype at the SLA locus exhibit complete penetrance. The major locus has the greatest effect on overall risk and may represent a tumour-initiator locus. The *B* haplotype at the SLA locus serves to modify the penetrance.

**Figure 1** Profile lod score function for assessing linkage between the unknown major locus and the SLA locus. The maximum likelihood estimate of the recombination fraction is noted by the arrow.

**Table III** Estimated penetrances for the most parsimonious two-locus model

| *Major locus genotype* | *SLA locus genotype* | *Frequency* | *Penetrance* |
|---|---|---|---|
| AA | XX | 0.080 | 0.000 ± 0.003 |
| AA | BX | 0.333 | 0.167 ± 0.043 |
| AA | BB | 0.349 | 0.372 ± 0.062 |
| Aa | XX | 0.057 | 0.753 ± 0.049 |
| Aa | BX | 0.134 | 1.000 ± 0.003 |
| Aa | BB | 0.031 | 1.000 ± 0.003 |
| aa | XX | 0.010 | 0.753 ± 0.049 |
| aa | BX | 0.005 | 1.000 ± 0.003 |
| aa | BB | 0.001 | 1.000 ± 0.003 |

### *Genetic effects on tumour burden*

Given our most parsimonious two-locus model allowing for gametic disequilibrium, we calculated the posterior probabilities of each two-locus genotype for each animal. These posterior probabilities were used to estimate the effects of the two loci on tumour burden, an index of severity. Figure 2 illustrates the tumour distribution in the 297 animals for

**Figure 2** Distribution of tumour burden, an index of severity, in 297 animals.

**Figure 3** Effects of the two-locus genotypes on quantitative tumour burden. Error bars indicate $\pm 1$ s.e.m.

whom the requisite data were available. Mean tumour burden was 1.25 in these animals. The observed distribution does not fit a Poisson distribution due to significant evidence for overdispersion (the variance is approximately three times larger than the mean), which may be the result of variation at the two hypothesised loci.

Figure 3 shows the relationship between tumour burden and two-locus phenotypes. Clearly, genetic variation has an important influence on this measure of disease severity. The two loci jointly explain a significant proportion ($21.2 \pm 2.9\%$) of the total variation in tumour burden.

### Discussion

We have established that two loci jointly determine the risk of SSCM in our pedigrees. Our unknown major locus may represent a tumour-initiator or -suppressor gene responsible for SSCM initiation. The other locus is found in (or co-segregates with) the SLA complex and is responsible for modifying penetrance at the initiator locus. Since the major histocompatability complex has an important role in the immune response of all mammalian species, our finding of an SLA association with risk of melanoma implies that research on immunological factors needs to be further pursued. Questions regarding whether a specific immune mechanism is involved in melanoma initiation also need to be addressed.

Unlike the genetic analysis of CMM in human pedigrees SSCM is not likely to express genetic heterogeneity since it arose in a single family within a genetically isolated herd. In this study all SSCM cases could be traced paternally to the descendants of a single founding boar or the male offspring of a single gilt. Additionally, our large swine pedigrees appear to be superior to most human pedigrees for resolving oligogenic forms of inheritance.

Having documented the two-locus inheritance of SSCM our next goal is to map the unknown major locus. Cytogenetic abnormalities in SSCM cell lines have been identified for chromosome regions 2p and 2q (syntenic to human chromosome 11p), 6q (syntenic to human chromosome 19q), 13 (syntenic to 3q and 13q) and 14 (syntenic to 8p and 10q) (Green *et al.*, 1992; Rohrer *et al.*, 1994) and suggest potential candidate regions for future linkage analyses. Our recent progress in porcine gene mapping has generated the most complete genetic linkage information for a livestock species to date (Rohrer *et al.*, 1994). Nearly 400 microsatellite loci in 24 linkage groups that cover approximately 2000 cM have been mapped. We intend to use these and additional microsatellite markers in a systematic genomic search for the tumour-initiator (-suppressor) locus.

**Acknowledgements**
This work was supported in part by grant 1PO1-CA49488 from the National Cancer Institute, NIH.

### References

BALE SJ, CHAKRAVARTI A AND GREENE MH. (1986). Cutaneous malignant melanoma and familial dysplastic nevi: evidence for autosomal dominance and pleiotropy. *Am. J. Hum. Genet.*, **38,** 188–196.

BALE SJ, DRACOPOLI NC, TUCKER MA, CLARK WH, FRASER MC, STANGER BZ, GREEN P, DONIS-KELLER H, HOUSMAN DE AND GREENE MH. (1989). Mapping the gene for hereditary cutaneous malignant melanoma – dysplastic nevus to chromosome 1p. *N. Engl. J. Med.*, **320,** 1367–1372.

BLANGERO J, MACCLUER JW, KAMMERER CM, MOTT GE, DYER TD AND MCGILL Jr HC. (1990). Genetic analysis of apolipoprotein A-I in two dietary environments. *Am. J. Hum. Genet.*, **47,** 414–428.

BLANGERO, J., WILLIAMS-BLANGERO S, KAMMERER CM, TOWNE B AND KONIGSBERG LW. (1992). Multivariate genetic analysis of nevus measurements and melanoma. *Cytogenet. Cell Genet.*, **59,** 179–181.

BONNEY G.E. (1986). Regressive logistic models for familial disease and other binary traits. *Biometrics*, **42,** 611–625.

CANNON-ALBRIGHT LA, GOLDGAR DE, WRIGHT EC, TURCO A, JOST M, MEYER LJ, PIEPKORN M, ZONE JJ AND SKOLNICK MH. (1990). Evidence against the reported linkage of the cutaneous melanoma-dysplastic nevus syndrome locus to chromosome 1p36. *Am. J. Hum. Genet.*, **46,** 912–918.

CANNON-ALBRIGHT LA, GOLDGAR DE, MEYER LJ, LEWIS CM, ANDERSON DE, FOUNTAIN JW, HEGI ME, WISEMAN RW, PETTY EM, BALE AE, OLOPADE OI, DIAZ MO, KWIATKOWSKI DJ, PIEPKORN MW, ZONE JJ AND SKOLNICK MH. (1992). Assignment of a locus for familial melanoma, MLM, to chromosome 9p13–p22. *Science*, **258,** 1148–1152.

DANES BS AND LYNCH HT. (1993). *In vitro* evidence for the miniature pig as an animal model for familial atypical multiple mole melanoma (FAMMM) syndrome. *Lab. Animal*, **12,** 42–44.

ELSTON RC AND STEWART J. (1971). A general model for the genetic analysis of pedigree data. *Hum. Hered.*, **21,** 523–542.

GREEN A AND SWERDLOW AJ. (1989). Epidemiology of melanocytic nevi. *Epidemiol. Rev.*, **11,** 204–221.

GREEN A, SHILKAITIS H, BRATESCU L, AMOSS Jr MS AND BEATTIE CW. (1992). Establishment and characterization of four Sinclair swine cutaneous malignant melanoma cell lines. *Cancer Genet. Cytogenet.*, **61**, 77-92.

GREENE MH, GOLDIN LR, CLARK WH, LOVRIEN E, KRAEMER KH, TUCKER MA, ELDER DE, FRASER MC AND ROWE S. (1983). Familial malignant melanoma: autosomal dominant trait possibly linked to the Rh locus. *Proc. Natl Acad. Sci. USA*, **80**, 6071-6075.

GRUIS NA, BERGMAN W AND FRANTS RR. (1990). Locus for susceptibility to melanoma on chromosome 1p. *N. Engl. J. Med.*, **322**, 853-854.

HASSTEDT SJ. (1989). Pedigree Analysis Package. V3.0 Department of Human Genetics, University of Utah.

HASSTEDT SJ AND MOLL PP. (1989). Estimation of genetic model parameters: variables correlated with a quantitative phenotype exhibiting major locus inheritance. *Genet. Epidemiol.*, **6**, 319-332.

HOOK Jr RR, AULTMAN MD, ADELSTEIN EH, OXENHANDLER RW, MILLIKAN LE AND MIDDLETON CC. (1979). Influence of selective breeding on the incidence of melanoma in Sinclair miniature swine. *Int. J. Cancer*, **24**, 668-672.

HOOK Jr RR, HAMBRY CV, MILLIKIN LE, BERKELHAMMER J AND STILLS Jr HF. (1983). Cell-mediated immune reactivity of Sinclair swine melanoma-bearing swine to 3MKC1 extracts of swine and human melanoma. *Int. J. Cancer*, **31**, 663-637.

JONES DH AND AMOSS Jr MS. (1982). Cell mediated immune response in miniature Sinclair swine bearing cutaneous melanomas. *Can. J. Comp. Med.*, **46**, 209-211.

LALOUEL JM. (1979). *GEMINI: a Computer Program for Optimization of a Nonlinear Function*, Tech. Rep. 14. Department of Medical Biophysics and Computing: University of Utah, Salt Lake City.

LALOUEL JM, RAO DC, MORTON NE AND ELSTON RC. (1983). A unified model for complex segregation analysis. *Am. J. Hum. Genet.*, **35**, 816-826.

MILLER RG. (1974). The jackknife: a review. *Biometrika*, **61**, 1-15.

MILLIKIN LE, HOOK RR AND MANNING PJ. (1973). Gross and ultrastructural studies in a new animal model of melanoma: the Sinclair swine. *Yale J. Biol. Med.*, **46**, 631-645.

NEUMAN R, VAN EERDEWEGH P, MOLDIN S AND ROCHBERG N. (1992). Genetic analysis of cutaneous melanoma and dysplastic nevi under varying phenotypic definitions. *Cytogenet. Cell Genet.*, **59**, 214-216.

ROHRER GA, ALEXANDER LJ, KEELE JW, SMITH TP AND BEATTIE CW. (1994). A micro-satellite linkage map of the porcine genome. *Genetics*, **136**, 231-245.

SCHWAB M. (1987). Oncogenes and tumor suppressor genes in *Xiphoporus*. *Trends Genet.*, **3**, 38-42.

SPEER MC, HAYNES CS AND PERICAK-VANCE MA. (1992). Segregation analysis in cutaneous malignant melanoma/dysplastic nevus syndrome families. *Cytogenet. Cell Genet.*, **59**, 225-227.

TISSOT RG, BEATTIE CW AND AMOSS Jr MS. (1987). Inheritance of Sinclair swine cutaneous malignant melanoma. *Cancer Res.*, **47**, 5542-5545.

TISSOT RG, BEATTIE CW AND AMOSS Jr MS. (1989). The swine leucocyte antigen (SLA) complex and Sinclair swine cutaneous malignant melanoma. *Anim. Genet.*, **20**, 51-57.

TISSOT RG, BEATTIE CW, AMOSS Jr MS, WILLIAMS JD AND SCHUMACHER J. (1993). Common swine leucocyte antigen (SLA) haplotypes in NIH and Sinclair miniature swine have similar effects on the expression of an inherited melanoma. *Anim. Genet.*, **24**, 191-193.